# Hemorrhagic Transformation: A Review of the Rate of Hemorrhage in the Major Clinical Trials of Acute Ischemic Stroke

**DOI:** 10.3389/fneur.2013.00069

**Published:** 2013-06-10

**Authors:** Eric S. Sussman, E. Sander Connolly

**Affiliations:** ^1^Cerebrovascular Laboratory, Department of Neurosurgery, Columbia University, New York, NY, USA

**Keywords:** acute ischemic stroke, hemorrhagic transformation, intracerebral hemorrhage, fibrinolysis, mechanical thrombectomy

## Abstract

Acute ischemic stroke is a devastating disease that is often complicated by hemorrhagic transformation. While significant advances have been made over the past two decades with regard to emergent treatment of AIS, many of the therapeutic options are limited by an increased risk of hemorrhage. Here, we sought to review the rates of hemorrhagic transformation in the major clinical trials of AIS intervention. Since the reviewed clinical trials vary significantly in terms of study design, eligibility criteria, stroke severity, and baseline demographic data, direct comparisons between the various trials is not valid. Nevertheless, this review is intended to consolidate the pertinent data on hemorrhagic transformation in order to call attention to any patterns that may warrant further investigation.

## Introduction

Intracerebral hemorrhage (ICH) is the most common and the least treatable subtype of hemorrhagic stroke (Sudlow and Warlow, 1997; Flaherty et al., 2005; Zahuranec et al., 2006; Broderick et al., 2007; Feigin et al., 2009; Sacco et al., 2009). Although the management of ICH is a topic of intense investigation, treatment options remain limited. Thus, there is a dire need for continued research into optimal ICH management. In addition, it is critical that efforts are simultaneously directed toward *preventing* ICH before it occurs. This requires a comprehensive understanding of the underlying etiology and pathophysiology of the disease. In this context, it is important to distinguish between spontaneous (non-lesional) ICH – parenchymal hemorrhage that occurs in the absence of an underlying lesion – and secondary ICH, which occurs in the setting of cerebral tumor, vascular malformation, ischemic injury, or other underlying lesion. A multitude of risk factors for spontaneous ICH have been identified, including but not limited to hypertension, cerebral amyloid angiopathy (CAA), sympathomimetic drugs, and advanced age (Harrington et al., 1983; Cosgrove et al., 1985; Hornig et al., 1986; Lowenstein et al., 1987; Zodpey et al., 2000; Ariesen et al., 2003). Yet, while such risk factors are overwhelmingly common in the general population (Vinters and Gilbert, 1983; Hajjar and Kotchen, 2003), only a small fraction of these patients experiences ICH. In contrast, secondary ICH is more predictable, particularly in the setting of an underlying ischemic injury. Hornig et al. (1986) observed radiographic evidence of hemorrhage in 43% (28/65) of cases of acute ischemic stroke (AIS) followed prospectively with serial computed tomography (CT) imaging for 4 weeks. Similarly, Okada et al. (1989) reported a 40.6% (65/160) rate of hemorrhage in a separate cohort of AIS patients followed prospectively with serial radiographic imaging. Thus, it is clear that *hemorrhagic transformation* occurs in a significant proportion of patients following ischemic brain injury.

In this review, we will evaluate the relevant literature regarding hemorrhagic transformation of acute ischemic stroke (AIS). First, we will provide a brief overview of the pathogenesis and classification of hemorrhagic transformation. In turn, we will review the incidence of hemorrhagic transformation in the major clinical trials of AIS over the past two decades.

## Pathophysiology of Hemorrhagic Transformation

Hemorrhagic transformation of acute ischemic stroke is a complex and multifactorial phenomenon. Within seconds to minutes after the onset of ischemia, there is a reduction in ATP and a subsequent cessation of Na^+^-K^+^ ATPase activity (Rossi et al., 2007). This leads to a variety of cellular and metabolic derangements, which collectively lead to a disruption of the blood-brain barrier (BBB) (Warach and Latour, 2004). Furthermore, ischemia results in a robust inflammatory response (Janardhan and Qureshi, 2004; Mocco et al., 2006; Komotar et al., 2008), which further disrupts normal cerebrovascular anatomy and physiology. The resulting disruption of the BBB along with the impairment of autoregulatory capacity of the cerebral vasculature predisposes to blood extravasation when the ischemic tissue is eventually reperfused (Khatri et al., 2012). Importantly, it appears that the degree of anatomical and physiological disruption is highly dependent on the duration of ischemia (Rossi et al., 2007; Khatri et al., 2012).

## Classification of Hemorrhagic Transformation

There is a broad spectrum of severity of hemorrhagic transformation, ranging from subtle petechial hemorrhage within infarcted tissue to large-volume hematoma extending beyond the borders of the infarction (Hornig et al., 1986). The term *hemorrhagic infarction* (HI) describes heterogeneous hyperdensity occupying a portion of an ischemic infarct zone on CT-imaging, whereas *parenchymatous hematoma* (PH) refers to a more homogeneous, dense hematoma with mass effect (Pessin et al., 1990; del Zoppo et al., 1992). Fiorelli et al. (1999) refined these criteria to include two subtypes of HI (HI1 and HI2) and two subtypes of PH (PH1 and PH2), reflecting the broad spectrum of hemorrhagic transformation (Table 1).

**Table 1:** **Radiographic classification of the spectrum of hemorrhagic transformation, based on criteria proposed by Fiorelli et al. (1999)**.

Hemorrhage classification	Radiographic appearance
Hemorrhage infarction type 1 (HI1)	Small hyperdense petechiae
Hemorrhage infarction type 2 (HI2)	More confluent hyperdensity throughout the infarct zone; without mass effect
Parenchymal hematoma type 1 (PH1)	Homogeneous hyperdensity occupying<30% of the infarct zone; some mass effect
Parenchymal hematoma type 2 (PH2)	Homogeneous hyperdensity occupying>30% of the infarct zone; significant mass effect. Or, any homogenous hyperdensity located beyond the borders of the infarct zone

In addition to providing a framework for characterizing hemorrhage after AIS, these criteria may be predictive of the time course as well as the clinical significance of hemorrhagic transformation. In the study by Hornig et al. (1986) described previously, hemorrhages that occurred within the first week after AIS onset were more likely to take the form of dense hematoma (consistent with PH2-type hemorrhagic transformation), as opposed to hemorrhages occurring at more distant time points after AIS onset, which were more likely to be HI1, HI2, or PH1. In a *post hoc* analysis of the European Cooperative Acute Stroke Study I (ECASS-I) – a randomized, placebo-controlled phase III clinical trial of recombinant tissue plasminogen activator (rtPA) in AIS – PH2-type hemorrhagic transformation was found to be a significant predictor of neurological deterioration (OR 32.3; 95% CI 13.4–77.7) and of 3 month mortality (OR 18.0; 95% CI 8.1–40.1), whereas HI1, HI2, and PH1 were not associated with either increased morbidity or mortality (Fiorelli et al., 1999). Despite the utility of this detailed classification system, many studies characterize hemorrhagic transformation as simply *symptomatic* or *asymptomatic* (Multicentre Acute Stroke Trial – Italy (MAST-I) Group, 1995; The National Institute of Neurological Disorders, 1995; The Multicenter Acute Stroke Trial – Europe Study Group, 1996; The NINDS t-PA Stroke Study Group, 1997).

## Rates of Hemorrhagic Transformation in Clinical Trials of AIS

Over the past two decades, a variety of treatment strategies for AIS have become available. While the potential benefit is unquestionable, these treatment options also have inherent risks of their own. Fibrinolytic agents – including rtPA and prourokinase – may increase the risk of systemic and intracranial hemorrhage by nature of their potent fibrinolytic activity. Alternatively, endovascular treatment modalities may result in mechanical damage to the blood vessel endothelium, thereby increasing hemorrhage risk. Thus, it is critical to weigh the potential benefits of vessel recanalization against the added risk of hemorrhage due to the particular therapy that is used. In the remainder of this manuscript, we will review the rates of hemorrhagic transformation in the major clinical trials of AIS (summarized in Table 2). It is important to emphasize that there is a significant degree of heterogeneity between the design and eligibility criteria of each of the clinical trials that will be discussed. As a result, the relative rates of hemorrhagic transformation cannot be directly compared, and any patterns that are identified must be further investigated in randomized controlled trials.

**Table 2:** **Summary of hemorrhagic transformation data from major clinical trials of AIS intervention**.

Clinical trial	Sample size	Duration of radiographic follow up	Asymptomatic hemorrhagic transformation rate	Symptomatic hemorrhagic transformation rate	Parenchymal hemorrhage type 2 rate	Time to treatment^+^
**IV FIBRINOLYSIS**						
NINDS	312	7–10 days	4.5% (14/312)	6.4% (20/312)	N/A	1.5 h^p^
ECASS-II	409	7 days	39.6% (161/407)	8.8% (36/407)	8.1% (33/407)	N/A
ATLANTIS	272	18–30 h	11.4% (31/272)	7.0% (19/272)	N/A	4.36 h^p^
SITS-MOST	6483	22–36 h	9.6% (617/6438)	7.3% (468/6483)	2.9% (184/6352)	2.3 h^p^
ECASS-III	418	36 h	27% (113/418)	2.4% (10/418)	1.9% (8/418)	3.98 h^p^
IST-III	1515	7 days	N/A	6.9% (104/1515)	N/A	4.2 h^p^
**IA FIBRINOLYSIS**						
PROACT-I	26	90 days	50% (13/26)	15.4% (4/26)	7.7% (2/26)	5.5 h^p^
PROACT-II	121	7–10 days	68% (73/108)	10% (11/108)	N/A	4.5 h^p^
**COMBINED IV/IA APPROACH**						
EMS	17	7 ± 1 days	11.8% (2/17)	23.5% (4/17)	0.0% (0/17)	2.6 h^p^/6.3 h^q^
IMS-I	80	36 h	43% (34/80)	6.3% (5/80)	7.5% (6/80)	1.33 h^p^/3.53 h^q^
IMS-II	81	36 h	32.1% (26/81)	9.9% (8/81)	8.6% (7/81)	2.37 h^p^/N/A^q^
IMS-III	434	24 ± 6 h	27.4% (119/434)	6.2% (27/434)	5.8% (25/434)	2.03 h*^p^/N/A^q^
**MECHANICAL THROMBECTOMY**						
Merci
Phase I	28	24 h	42.9% (12/28)	0.0% (0/28)	0.0% (0/28)	6.25 h^q^
MERCI Trial	141	24 h	27.7 (39/141)	7.8% (11/141)	1.4% (2/141)	6.4 h*^q^
Multi-MERCI	164	24 h	30.5% (50/164)	9.8% (16/164)	2.4% (4/164)	5.9 h*^q^
Penumbra
Pivotal Stroke Trial	125	24 h	16.8% (21/125)	11.2% (14/125)	1.6% (2/125)	4.85 h^q^
POST Trial	157	24 h	13.4% (21/157)	6.4% (10/157)	N/A	5.18 h^q^
Solitaire
Phase I	20	24 h	30% (6/20)	10.0% (2/20)	5.0% (1/20)	6.7 h^q^
SWIFT Trial	58	24 h	15.5% (9/58)	2.0% (1/58)	0.0% (0/58)	5.54 h*^q^
Trevo
TREVO	60	N/A	N/A	N/A	N/A	5.03 h^q^
TREVO-2	88	18–36 h	40.9% (36/88)	4.5% (4/88)	8.0% (7/88)	5.4 h*^q^

^+^ Expressed as median value, except when marked with an asterisk.

* Expressed as mean (only when relevant median value was not published).

^p^ Time to *initiation* of IV fibrinolytic therapy.

^q^ Time to clot lysis (or time to procedure termination when clot lysis was not achieved).

Duration of radiographic follow up expressed in hours (h) or days (d). Clinical trial acronyms are elaborated in the manuscript text.

### Intravenous fibrinolysis

In 1996, the United States Food and Drug Administration (FDA) approved intravenous (IV) rtPA for the treatment of AIS. The most significant concern surrounding the use of IV rtPA is an increased risk of hemorrhage, which is particularly concerning given the already significant rate of hemorrhagic transformation of AIS even in the absence of a potent fibrinolytic agent. Nonetheless, the National Institute of Neurological Disorders and Stroke (NINDS) rtPA Stroke Study demonstrated surprisingly low rates of both asymptomatic (4.5%) and symptomatic (6.4%) hemorrhagic transformation (4.5%) (The National Institute of Neurological Disorders and Stroke rtPA Stroke Study Group, 1995). The Safe Implementation of Thrombolysis in Stroke-Monitoring Study (SITS-MOST), a large observational study of IV rtPA, also demonstrated a relatively low rate of asymptomatic hemorrhage (9.6%) with an additional 7.3% of patients experiencing symptomatic hemorrhage (Wahlgren et al., 2007). On the other hand, the European Cooperative Acute Stroke Study (ECASS)-II and -III trials (Hacke et al., 1998, 2008) revealed much higher rates of asymptomatic hemorrhage (39.6 and 27%, respectively), which was more consistent with what had been reported in the literature in prior observational studies of AIS (Hornig et al., 1986; Okada et al., 1989).

One potential explanation for the discrepantly low asymptomatic hemorrhage rates in NINDS and SITS-MOST as compared with ECASS-II and ECASS-III is the difference in the time window during which the fibrinolytic drug was administered. More specifically, NINDS and SITS-MOST both required that the drug be administered within 3 h of AIS onset, whereas ECASS-II and -III evaluated drug treatment at later time points (within 6 h, and between 3 and 4.5 h, respectively). As described previously, the duration of ischemia is a critical determinant of the degree of vascular disruption, and so the delayed drug administration in the ECASS studies *may* account for the increased rate of hemorrhage. However, the ATLANTIS Trial (Clark et al., 1999), which also evaluated drug administration beyond the 3 h window, revealed hemorrhage rates more in-line with those found in NINDS and SITS-MOST. Thus, it is likely that the variation in hemorrhage rates between the different trials is multifactorial – differences in eligibility criteria and/or in the way hemorrhage was defined in each of the trials may have contributed to this variation.

Although our intention is to review the rates of hemorrhagic transformation in the major *clinical trials* of AIS intervention, it is also worthwhile to consider the incidence of hemorrhage in clinical practice with IV rtPA. Graham (2003) performed a meta-analysis of 15 open-label studies consisting of 2639 patients treated with IV rtPA in the non-clinical trial setting; this analysis revealed a symptomatic hemorrhage rate of 5.2%, in addition to a 6.3% asymptomatic hemorrhage rate. Each of the included trials conformed to the approved 3 h time window for drug administration and included a broad range of patient demographics and stroke severities, as encountered in clinical practice.

### Intraarterial fibrinolysis

The intraarterial (IA) route of fibrinolytic drug delivery provides direct access to the occluded vessel and thereby may reduce the dose of fibrinolytic agent that is required. In turn, this may decrease the risk of hemorrhagic transformation. Alternatively, an IA approach to drug delivery may in fact increase hemorrhage rates due to increased exposure of the injured tissue to the fibrinolytic agent. In other words, the infarct zone is exposed to the full dose of fibrinolytic drug when administered intraarterially, whereas when administered intravenously, the extent of drug exposure in the area of ischemic injury is dependent on the proportion of cardiac output that reaches that particular vascular territory. In PROACT-I and PROACT-II – phase II and III clinical trials of IA prourokinase – the rates of asymptomatic hemorrhage were 50 and 68%, respectively, while the rates of symptomatic hemorrhage were 15.4 and 10%, respectively (del Zoppo et al., 1998; Furlan et al., 1999). In comparison to the rates observed in clinical trials of IV fibrinolysis, IA drug administration appears to be associated with higher rates of both symptomatic and asymptomatic hemorrhage. This may be partially due to inevitable delays in treatment that occur during mobilization of resources for endovascular intervention, however a multitude of additional factors are likely to contribute to this discrepancy as well.

It is important to note that this is not an exhaustive review of the clinical trials that have evaluated IA fibrinolysis for AIS. Certain large, well-designed clinical trials – such as MELT Japan (Ogawa et al., 2007) – were not included due to restrictive inclusion criteria (i.e., middle cerebral artery occlusions only in the case of MELT) and/or failure to report asymptomatic hemorrhage rates.

### Combined intravenous and intraarterial approaches

More recently, some specialized stroke centers have adopted a combined IV and IA approach, in which patients are treated with IV fibrinolysis during transport to the endovascular suite for further IA intervention. This approach affords the advantages of direct access to the occluded vessel without the inherent delay in treatment associated with an exclusively endovascular intervention. However this approach also entails increased exposure to fibrinolytic agents and endovascular manipulation and thus a theoretically increased risk of hemorrhagic transformation. Combined approached have been evaluated in a number of clinical trials, including the Emergency Management of Stroke (EMS) Bridging Trial (Lewandowski et al., 1999), as well as the Interventional Management of Stroke (IMS)-I, -II, and -III Trials (IMS Study Investigators, 2004; IMS-II Trial Investigators, 2007; Broderick et al., 2013). The rates of asymptomatic hemorrhage in EMS, IMS-I, IMS-II and IMS-III were 11.8, 43, 32.1, and 27.4%, respectively, whereas symptomatic hemorrhagic transformation occurred in 23.5, 6.3, 9.9, and 6.2%, respectively.

Although the rate of asymptomatic hemorrhage was comparatively low in EMS, this was compensated for by an anomalously high rate of *symptomatic* hemorrhage. Interestingly, the rate of symptomatic hemorrhage in the combined IV/IA fibrinolysis group was similar to that in the control group, which received IV placebo prior to IA fibrinolysis. Thus, the high rate of symptomatic hemorrhage in this trial may be a reflection of the eligibility criteria and/or the trial protocol, rather than a reflection of the combined IV/IA approach. In addition, EMS was a small phase I trial that was not adequately powered to detect an increased incidence of hemorrhagic complications, and so generalized conclusions about the hemorrhage rate with combined IV/IA fibrinolysis should not be drawn based solely on the results of this trial. On the other hand, the hemorrhage rates in the IMS trials were lower than those seen in PROACT studies, thus suggesting that the combined approach is at least as safe as an exclusively IA approach; however, it is important to reemphasize that caution must be exercised when comparing disparate clinical trials with distinct eligibility criteria and study designs.

### Mechanical thrombectomy

Mechanical thrombectomy is an endovascular approach to AIS treatment that attempts to remove the occluding thromboembolus en bloc from the blood vessel. One theoretic advantage of mechanical thrombectomy is that it may allow for a reduction in the required dose of fibrinolytic, or perhaps may even eliminate the need for fibrinolysis altogether. In other words, mechanical thrombectomy may be used as an alternative to fibrinolytic treatment, or as an adjunct to fibrinolysis in order to achieve higher rates of recanalization than either treatment strategy alone.

The Merci Retriever (Concentric Medical, Mountain View, CA, USA) was the first mechanical thrombectomy device that received FDA-approval for use in AIS patients. In the phase I trial of this device (Gobin et al., 2004), asymptomatic hemorrhagic transformation occurred in 42.9% (12/28) of these patients, but there were no cases of symptomatic hemorrhage. In the similarly designed phase II trial (Smith et al., 2005), 141 patients were treated with the Merci Retriever. Amongst these patients, the rates of asymptomatic and symptomatic hemorrhagic transformation were 27.7 and 7.8%, respectively. While it is noteworthy that there were zero cases of symptomatic hemorrhage in the phase I trial, the phase II trial revealed a similar rate of symptomatic hemorrhage as had been seen in prior trials of fibrinolytic agents, thus suggesting that the results of the phase I trial may be attributable to the small sample size. A separate prospective trial of the Merci Retriever (Smith et al., 2008), which included patients with refractory vessel occlusion following IV rtPA administration, demonstrated comparable rates of both asymptomatic and symptomatic hemorrhagic transformation (30.5 and 9.8%, respectively), thus suggesting that prior administration of IV rtPA does not significantly increase the hemorrhage risk associated with the use of the Merci device.

The next endovascular device to receive FDA-approval for use in AIS patients was the Penumbra Thromboaspiration System (Penumbra, Alameda, CA, USA). The FDA’s approval of this device was largely based on the results of the Penumbra Pivotal Stroke Trial (Penumbra Pivotal Stroke Trial Investigators, 2009), which revealed significantly higher rates of vessel recanalization than any previously evaluated treatment for AIS, with a comparatively low rate of asymptomatic hemorrhagic transformation (16.8%) and a comparable rate of symptomatic hemorrhage (11.2%), relative to the trials of the Merci Retriever or fibrinolytic agents. Importantly, statistical analysis revealed that adjunctive use of fibrinolytic therapy (either IV or IA) was not a significant predictor of hemorrhagic transformation in this study. The POST Trial (Tarr et al., 2010) – a phase IV post-marketing surveillance study of the Penumbra device – confirmed the low rates of both asymptomatic and symptomatic hemorrhage (13.4 and 6.4%, respectively).

One of the newest classes of mechanical thrombectomy devices – reversible stents, or stent retrievers – has come into favor as a result of high rates of vessel recanalization. There are two FDA-approved stent retrievers for use in AIS patients: the Solitaire Flow Restoration Device (ev3, Plymouth, MN, USA) and the Trevo Retriever (Concentric Medical, Mountain View, CA, USA). Twenty patients were enrolled in the phase I trial of the Solitaire device (Castaño et al., 2010), of which 30% were found to have asymptomatic hemorrhagic transformation and 10% had symptomatic hemorrhage. In the subsequently performed SWIFT Trial (Saver et al., 2010), a randomized, parallel-group non-inferiority study comparing the Solitaire Flow Restoration Device to the Merci Retriever, 58 patients were treated with the Solitaire device, with only one patient (2.0%) experiencing symptomatic hemorrhagic transformation, and an additional 15.5% with asymptomatic hemorrhage. The SWIFT Trialists attribute these low hemorrhage rates to the mechanical superiority of the Solitaire device; more specifically, they posit that the Solitaire device exerts less radial pressure on the blood vessel as compared with other mechanical thrombectomy devices, thereby minimizing injury to the vessel endothelium. Nonetheless, while the hemorrhage rates within the Solitaire group of the SWIFT Trial were numerically lower than the equivalent rates in the Merci comparison group, this difference was not statistically significant (difference −9.2%; 95% CI −27.0–9.6; *p*-value 0.057).

The TREVO study was a phase II clinical trial of the Trevo Retriever in AIS patients. The results of this trial, which were presented at the 2012 International Stroke Conference in New Orleans, Louisiana (Wahlgren et al., 2012), demonstrated a high rate of vessel recanalization, but unfortunately the rate of hemorrhagic transformation was not reported. TREVO-2 (Nogueira et al., 2012) was a randomized, parallel-group phase III clinical trial that directly compared the Trevo device to the Merci Retriever. Of the 88 patients enrolled into the Trevo arm, 4.5% experienced symptomatic hemorrhagic transformation, while asymptomatic hemorrhage occurred in an additional 40.9%.

## Discussion

Acute ischemic stroke is associated with significant morbidity and mortality, and unfortunately this disease is often followed by subsequent hemorrhagic transformation, which may further complicate an already devastating clinical condition. While tremendous advances have been made with regard to emergent AIS management, an increased risk of hemorrhage remains an important limitation of many of the available therapeutic options. In this manuscript, we sought to review the rates of hemorrhagic transformation in the major clinical trials of AIS interventions. As mentioned previously, direct comparison of hemorrhage rates between the different trials is misleading; differences in clinical trial design, eligibility criteria, baseline stroke severity, and patient demographics all have the potential to confound such an analysis. Thus, the intention of this review is not to draw any definitive conclusions regarding the relative risk of hemorrhage with any particular treatment strategy or device, but rather to integrate the relevant data from multiple trials in order to call attention to any trends that may warrant further investigation.

We observed a trend toward increased hemorrhage rates as the time between stroke onset and vessel recanalization increases. More specifically, the lowest rates of hemorrhagic transformation were observed in trials of IV fibrinolytic agents, particularly when the drug was administered within 3 h of AIS onset. Meanwhile, hemorrhage rates were higher when the initial treatment approach was IA, which inevitably involved delays as the resources necessary for an endovascular intervention were mobilized. There are a number of potential explanations for this observation. It is possible that the increased time lapse prior to blood vessel recanalization may have a direct effect on rates of hemorrhagic transformation. In support of this hypothesis is the fact that studies that allowed for IV fibrinolytic administration beyond 3 h after symptom onset tended to result in higher rates of hemorrhagic transformation, as compared with IV fibrinolysis studies in which drug was administered within the 3 h window. Furthermore, this hypothesis is also consistent with the underlying pathophysiology of ischemic cerebrovascular injury: as described previously, inflammatory processes are initiated within seconds of the onset of ischemia, and this inflammation leads to a progressive disruption of the BBB. Therefore, as the amount of time until recanalization increases, the disruption of the BBB becomes more extensive, and concurrently the risk of hemorrhagic transformation becomes more significant. Another potential explanation is that endovascular interventions may be intrinsically associated with higher hemorrhage rates, perhaps as a result of mechanical manipulation of the blood vessels.

On the other hand, it is possible that confounding variables may be largely responsible for the trends observed in this review. As discussed above, these clinical trials vary significantly with regard to eligibility criteria, patient demographics, baseline stroke severity, and study design. Furthermore, the clinical trials discussed here were completed over a span of more than two decades. During that time period, there have been significant advances in many aspects of stroke management, including emergency response protocols, neurocritical care, and imaging technology. The fact that the more recently performed trials of endovascular thrombectomy have revealed significantly higher rates of *asymptomatic* hemorrhage (while *symptomatic* hemorrhage rates have remained fairly constant) supports the hypothesis that improved imaging technology may allow for the identification of subclinical hemorrhages that were simply not able to be identified in earlier trials of IV fibrinolysis. Thus, randomized controlled trials controlling for these confounding factors are imperative in order to evaluate the relative risk of hemorrhagic transformation with each of the available AIS treatment modalities.

In summary, this review of hemorrhagic transformation in the major clinical trials of AIS intervention demonstrates observable trends toward increasing hemorrhage rates with endovascular interventions, as well as with increased time lapses between stroke onset and vessel recanalization; randomized controlled trials are warranted to verify these observations. In addition, further investigation is necessary in order to identify other risk factors and precipitants for hemorrhagic transformation in the setting of AIS.

## Conflict of Interest Statement

The authors declare that the research was conducted in the absence of any commercial or financial relationships that could be construed as a potential conflict of interest.
